# Report on the Memoirs Presented to the Society of Pharmacy at Paris, in Consequence of the Prizes Offered in the Yeær 1809

**Published:** 1810-09

**Authors:** M. Bouillon Lagrange


*Report on the Memoirs presented to the Society of Pharmacy at Paris, in consequence of the Prizes offered in the Year* 1809. *Extracted by* M. Bouillon Lagrange *from the full Report drawn up by Messrs.* Nachet, Derosne, *and* Vallee.

Of nine Memoirs sent to the Society, two have particularly fixed the attention of the committee. They were written in answer to the following question:

"To prepare the acetate of potash, so as to obtain it white and saturated, without employing radical vinegar, and without having recourse to fusion; to point out which of the two, the acid or the alkali, gives it the colouring principle."

The first Memoir, with the motto *Ex cognitis incognita,* is written with great precision.

The author, after having ascertained the advantage which would result from obtaining this salt in all its purity by a simple and œconomical process, begins by inquiring from whence the colouring principle arises: it cannot, he says, be owing to the alkali, when it is considered that the fusion of the acetate of potash renders it insoluble, and that the heat requisite for this fusion is not so strong as that which is necessary for the preparation of any given potash; and on the other hand, it cannot be essential to the acetic acid, when radical vinegar is capable of instantly ly furnishing a colourless salt. Consequently, this colouring principle must be a foreign substance contained in common vinegar, and which may be introduced into it in distillation. But this same principle is less volatile than the acetic acid, since distilled vinegar leaves a residue of it if we evaporate it a second time: it is not very soluble by itself, and it cannot be dissolved except by the addition of acetic acid, since it is precipitated, at least in part, when we saturate the latter by potash: and finally, it has been ascertained that it is of a vegeto-animal nature, either from the smell which it exhales when placed on hot coals, or by the prussiate of ammonia, which upon distillation furnishes the acetate of potash prepared with distilled vinegar: a product which does not give the same salt prepared with radical vinegar: whence the author concludes that the radical principle which colours the acetate of potash is nothing but a part of the ferment of common vinegar, carried into the distillation and more or less altered by this operation.

Independently of this colouring principle, inherent in the constitution of common vinegar, the author of the Memoir mentions another still more capable of making the acetate of potash look brown: this is the empyreumatic oil with which the vinegar is charged when the distillation is carried too far. He further says, that this salt may also be coloured by the oxides of iron and of manganese contained in the alkali, or by the metallic utensils used in its preparation: but this colour being merely accidental, we may avoid it entirely by using a pure potash and vessels of tin or porcelain. We must therefore adhere to the ferment and the empyreumatic oil. The following directions are given for avoiding these two colouring principles: the ferment may be separated from the acetate of potash the more easily the less of it there is in the distilled vinegar, and the latter will contain so much the less in its turn; as in common vinegar, the proportion of the ferment will be smaller with respect to that of the acid, on account of the quantity of ferment brought over in distillation being always more or less in proportion with that which exists in common vinegar. It follows therefore that it is necessary, above all, to employ common vinegar, which is at once the most acid, and the least charged with ferment; and this requisite may be attained by choosing a clear vinegar, besides being very strong and completely fermented. After the choice of the vinegar, the process of distillation may also have some influence on the quantity of the ferment contained in distilled vinegar: for, since this principle is less volatile than the acetic acid, the less of it will pass over in distillation, the more slowly this process is conducted; and in this respect we may admit a slight ebullition as being the fittest degree of heat.

If the preceding rules have been well attended to, the distilled vinegar will contain so small a quantity of ferment that it will be capable of furnishing immediately an acetate of potash almost entirely colourless; but if, notwithstanding every precaution, the whiteness of the salt still leaves something to be desired, there remains a final method of remedying it, which consists in filtering through charcoal in powder. The action of this substance, although little known as to its theory, is nevertheless certain in its effects; since it is sufficient to boil slightly with it the solution of the acetate as above prepared, in order to obtain it perfectly white after filtration and evaporation carefully managed. As to the empyreumatic oil, there is only one way of avoiding it, which is to stop the distillation of the vinegar at the moment when this principle begins to come over, and the product gives out an empyreumatic smell: for, beyond this term, the vinegar, if still white in appearance, would not undergo any change of colour during the evaporation of the acetate; and this colour, when once produced, could not be removed, either by charcoal powder or by any other means whatever.

The second Memoir presents fuller details. Its motto is taken from Boileau:

> "L'artifice agréable

> Du plus affreux objet fait un objet aimable."

The author describes in the first instance the various processes hitherto adopted in preparing the acetate of potash. He mentions as the most exact the process of M. Bouillon Lagrange, which consists in crystallizing this salt: but he regrets not having been able to put it in practice, from the difficulty of separating the crystallized acetate from the mother waters, which are very thick. In order to obtain as advantageous a result by a more practicable process, he tried double decompositions; he treated acetate of lime with the carbonate or sulphate of potash, but he did not obtain an acetate of potash less coloured than if he had directly saturated the carbonate of potash with distilled vinegar.

It would be necessary, as he observes, to employ a crystallized acetate of lime, but in this case the process would become too tedious and expensive. The decomposition of the common acetate of lead by the carbonate of soda, furnished him with a tolerably white acetate of potash: although this method unites with the facility of using it, the advantage of being cheaper, the author of the memoir does not think it right to resort to it, because the smallest negligence in the operation may change a wholesome medicine into a deadly poison. Recuring to the combination described of distilled vinegar and potash, he first inquires, whence arises the colour assumed by this salt during its evaporation: he is also aware that it is owing to a foreign principle contained in the distilled vinegar; but he afterwards saw that this substance was very slightly of a colouring nature by itself: he observed that the acetate of potash well saturated, is found as a consequence of its evaporation with an excess of alkali; and it is this excess of alkali which reacts on the foreign principle contained in the distilled vinegar, and colours it. In order to show more clearly this reaction of the potash, he divided into two equal portions a solution of acetate of potash, he evaporated both at the same degree of heat, maintaining constantly in the one an excess of acid, and in the other an excess of alkali: the salt produced by the liquor with excess of acid was much less coloured than that furnished by the liquor with an excess of alkali*. After having ascertained the origin of the colouring principle and the cause which developes it, the author next endeavoured to destroy it; and charcoal, in his opinion, is the fittest agent: with this view, he filters the distilled vinegar through charcoal, he then saturates it with carbonate of potash, leaving in it an excess of acid, which he takes care to keep constantly in the liquor during its evaporation. The result is an acetate equally white with that obtained by means of fusion.

This process, although very simple, did not appear to him to be practicable, because the acetate of potash is mixed with a certain quantity of acetate of lime, to which

R 2 the

* We have reason to believe, from our own experiments, that the potash still reacts, but much less on the colouring principle, even when the liquor contains an excess of acid; since by operating in this manner we always obtain an acetate of potash which is more or less coloured, whilst the same vinegar is capable of furnishing acetate of lime, magnesia, and alumine, which are very white. Soda did not appear to us to act so strongly as potash on this principle.—*Note by the Committee.*

the lime contained in the charcoal has given rise; and this salt, by altering the purity of the acetate of potash, retards its desiccation. It would, indeed, be very easy to separate it by adding a slight excess of carbonate of potash, in order to precipitate the lime; and we should afterwards put in an excess of acid: but it is easier to saturate the acid first. The following is the process as described in the memoir:

Pour into distilled vinegar a solution of carbonate of potash, until no more carbonic acid is extricated: afterwards evaporate the liquor, taking care always to keep an excess of acid in it: when it is reduced to three-fourths, allow it to cool, in order to separate from it the sulphate of potash and some impurities; decant it in order to heat it, and pour it when hot on a charcoal filter.*

If the filtered liquor does not contain more free acid, add a little distilled vinegar; † then evaporate to dryness; and if we wish to obtain the acetate of potash well cleaned, we must, at the end of the evaporation, manage the fire properly, and not stir it; but in this case it is not so white as when we separate it with a silver spatula, and throw it on the edges of the basin as fast as it is formed at the surface of the liquid: this salt will also be whiter if dried by small portions.

On exposing for about twenty days to the solar rays the liquor filtered over charcoal, the author obtained a salt much whiter: hence he thinks that the same result might be obtained, by exposing to the light an acetate of potash made from distilled vinegar, without being filtered through charcoal.

He regrets that he has been unable to collect some important facts relative to the colouring matter: he remarked that it was partly precipitated after saturation; that it is a little soluble in water, and that a portion remains in solution in the liquid acetate of potash; that after having filtered distilled vinegar through very pure charcoal, like that which is produced from crystalized sugar, we no longer

---

* The acetate of lime when dried is less deliquescent than the acetate of potash, and yet it is much more difficult to produce the desiccation of it.—*Note by the Author.*

† A little acetic acid (radical vinegar) would be preferable; very little is necessary when care has been taken to filter the liquor in the neutral state: we must also take care that it is not acid, in consequence of the lime which is in the charcoal.

longer obtain, on saturating it with crystalized carbonate of potash, the same precipitate as before filtration.

The author of the memoir concludes, therefore,—

1. That the colouring matter of the acetate of potash belongs to a vegetable substance contained in distilled vinegar.

2. That this colouring matter is destroyed by charcoal.

3. That an excess of alkali, when we evaporate the result of the saturation of distilled vinegar by potash, may influence the whiteness of the acetate of potash.

4. That in order to obtain the salt pure white and saturated, it is sufficient to filter a concentrated solution of it over a small quantity of charcoal in powder; to keep in it afterwards to the end of the evaporation an excess of acid, by adding from time to time distilled vinegar, and to expose it for some days to the solar light.

In a note which terminates this memoir, the author says, that according to the valuable observation of Messrs. Vauquelin, Pontier and Derosne, he obtained two hectogrammes of excellent acetic ether, by rectifying over potash the first products of the dislation of 70 litres of distilled vinegar.

The committee carefully repeated the experiments detailed in the above memoirs. If we except the whitening effects on exposing the acetate to the sun, which did not succeed with them, they pronounced them to be all correct; indeed, the principal agent in the whitening process was already known. Lowitz has recommended the use of charcoal, in order to obtain an acetate of potash less coloured than by the ordinary process; but whether he has not sufficiently described the method of using it, or employed vinegar of a bad quality, ill distilled or ill saturated, over which the depurating qualities of the charcoal had no influence, several chemists have been unsuccessful.

From these considerations, and particularly from the satisfactory results obtained by the committee, they think themselves warranted in concluding, that the authors of the two memoirs would have done better by making known the principle and the causes of the colouring of this salt, at the same time that they indicated the means of preventing and removing them.

By following carefully the rules which they prescribe, and by taking all the precautions which they point out, we shall easily obtain, without having recourse to fusion, an acetate of potash very white and perfectly saturated.

 The

The society has therefore decreed a gold medal of the value of 100 francs to each of the authors of the memoirs.

The author of the memoir first mentioned is M. Bernoully of Bâle; and of the second, M. Fremy of Versailles.

